# Empowering Mental Health Monitoring Using a Macro-Micro Personalization Framework for Multimodal-Multitask Learning: Descriptive Study

**DOI:** 10.2196/59512

**Published:** 2024-10-18

**Authors:** Meishu Song, Zijiang Yang, Andreas Triantafyllopoulos, Zixing Zhang, Zhe Nan, Muxuan Tang, Hiroki Takeuchi, Toru Nakamura, Akifumi Kishi, Tetsuro Ishizawa, Kazuhiro Yoshiuchi, Björn Schuller, Yoshiharu Yamamoto

**Affiliations:** 1 Educational Physiology Laboratory Graduate School of Education The University of Tokyo Tokyo Japan; 2 Technical University of Munich Munich Germany; 3 Hunan University Changsha China; 4 Google San Carlos, CA United States; 5 Osaka University Osaka Japan; 6 Imperial College London London United Kingdom

**Keywords:** multimodal, multitask, daily mental health, mental health, monitoring, macro, micro, framework, personalization, strategies, prediction, emotional state, wristbands, smartphone, mobile phones, physiological, signals, speech data

## Abstract

**Background:**

The field of mental health technology presently has significant gaps that need addressing, particularly in the domain of daily monitoring and personalized assessments. Current noninvasive devices such as wristbands and smartphones are capable of collecting a wide range of data, which has not yet been fully used for mental health monitoring.

**Objective:**

This study aims to introduce a novel dataset for personalized daily mental health monitoring and a new macro-micro framework. This framework is designed to use multimodal and multitask learning strategies for improved personalization and prediction of emotional states in individuals.

**Methods:**

Data were collected from 298 individuals using wristbands and smartphones, capturing physiological signals, speech data, and self-annotated emotional states. The proposed framework combines macro-level emotion transformer embeddings with micro-level personalization layers specific to each user. It also introduces a Dynamic Restrained Uncertainty Weighting method to effectively integrate various data types for a balanced representation of emotional states. Several fusion techniques, personalization strategies, and multitask learning approaches were explored.

**Results:**

The proposed framework was evaluated using the concordance correlation coefficient, resulting in a score of 0.503. This result demonstrates the framework’s efficacy in predicting emotional states.

**Conclusions:**

The study concludes that the proposed multimodal and multitask learning framework, which leverages transformer-based techniques and dynamic task weighting strategies, is superior for the personalized monitoring of mental health. The study indicates the potential of transforming daily mental health monitoring into a more personalized app, opening up new avenues for technology-based mental health interventions.

## Introduction

### Background

Mental health, recognized as a critical component of overall well-being, has garnered increasing attention and concern. The World Health Organization [1] defines mental health as a state of well-being where individuals realize their potential, cope with normal life stresses, work productively, and contribute to their community. However, mental health issues continue to present a significant burden globally, affecting individuals’ quality of life and posing challenges. In response to these challenges, the concept of “daily mental health monitoring” has emerged as a critical area of research and application [2,3]. This concept refers to the regular, continuous observation and assessment of an individual’s emotional states, using a variety of methods and tools to capture data in real time [4]. Such monitoring aims to provide a comprehensive understanding of an individual’s mental health, facilitating early detection of patterns, changes, or emerging issues. Consequently, accurate monitoring and understanding of daily mental health have become imperative for timely interventions and sustained mental well-being.

However, the field of daily mental health monitoring remains surprisingly underdeveloped, particularly regarding real-life applications [4]. The challenges faced by existing datasets and methods are not merely academic concerns but represent significant barriers to the effective and widespread adoption of mental health monitoring in everyday life. These challenges include the following.

- Real-world representation where a significant portion of existing datasets lack data derived from real-world settings, instead relying on artificial or laboratory conditions.
- Lack of self-annotation. many datasets do not use self-annotation [5-8], relying instead on experts’ observation or clinical interpretation. This approach often fails to capture the subjective experience of the individual, crucial for a person-centered understanding and monitoring of mental health [4,9]. In addition, clinical assessments typically occur at discrete time points, potentially missing the dynamic, moment-to-moment fluctuations in mental states that individuals experience in their daily lives [10].
- Challenges in accessibility of monitoring data. Many studies use electroencephalography (EEG) [11,12] while providing valuable insights into brain activity and emotional states, requiring specialized equipment and expertise, making it impractical for daily monitoring. Similarly, facial expression data [13,14] capture often necessitates continuous video monitoring, posing substantial privacy and practicality challenges for everyday use.
- Limited modalities and single-model approach. Most available research focuses on a single modality [15,16], this overlooks the inherently multimodal nature of human emotional expression and mental states, reducing the systems’ reliability.

To address the aforementioned challenges, our study adopts an innovative methodology aimed at forging more accurate, and efficacious tools for mental health monitoring. The contributions of our research are manifold, highlighted by the following key developments.

- Introducing a novel dataset collected from 298 individuals using noninvasive, everyday devices including wristband-type devices and smartphones, our dataset captures physiological signals: zero crossing mode (ZCM), proportional integration mode (PIM), and speech data. Participants provided self-annotated emotional states over 2 weeks, creating a rich, multimodal resource for understanding daily mental health dynamics.
- Developing a macro-micro framework for personalized daily mental health. Our framework develops a multimodal and multitask learning (MTL) strategy, innovatively built global emotion embeddings with individual personalization embedding.

In our research, the decision to focus on physiological signals and speech data, while excluding modalities such as facial expressions and EEG, was driven by several key considerations: Physiological signals have been shown to have a significant association with mental health and well-being. These signals, such as heart rate, skin conductance, and activity levels, can provide valuable insights into an individual’s emotional and psychological state [17]. The relationship between physiological signals and mental health is complex and multifaceted. For example, changes in heart rate variability (HRV) have been linked to stress, anxiety, and depression [18]. Reduced HRV has been observed in individuals with mental health disorders, suggesting that it may serve as a potential biomarker for mental well-being [19].

In our research, we apply the wrist-worn device used in our study which is equipped with a highly sensitive piezoelectric accelerometer that can detect even the most subtle wrist movements, with a resolution as fine as 0.01 G/rad/s. This allows for the capture of a wide range of daily activities and movements that may be relevant to mental health assessment [20]. The device uses 2 key modes for processing the accelerometer data: ZCM and PIM [21].

In addition to physiological signals, our study also incorporates speech data as a key modality for assessing mental health. Speech provides a rich source of information about an individual’s emotional state, cognitive functioning, and overall well-being [22]. There are several reasons why speech is a valuable tool for mental health assessment. First, speech carries emotional information through various features such as tone, pitch, and intonation. Changes in these features can reflect an individual’s emotional state, such as increased monotonicity in speech being associated with depression [23]. Second, speech patterns and characteristics can provide insights into an individual’s cognitive processes. For example, changes in speech fluency, coherence, and word choice have been linked to cognitive impairments and mental health conditions [24]. In addition, speech data can be collected noninvasively using readily available devices such as smartphones or voice recorders. This makes it a convenient and accessible modality for mental health assessment, especially in remote or telehealth settings [25].

Ecological momentary assessment (EMA) is a key methodological approach used in our study. EMA involves the repeated sampling of individuals’ current behaviors and experiences in real time, in their natural environments [26]. While EMA offers several advantages, such as reducing recall bias and capturing the dynamics of mental states in real-world contexts [10], it also has limitations. These include potential reactivity (ie, the act of self-reporting influencing the very experiences being reported) and compliance issues [27]. In this study, we aim to mitigate these limitations through careful design and participant training, which will be discussed in the Methods section.

Furthermore, our study introduces the Dynamic Restrained Uncertainty Weighting (DRUW) fusion method, a novel approach for integrating multimodal data in the context of mental health monitoring. The DRUW fusion method adaptively weights the contribution of each modality based on its uncertainty and distinct characteristics, ensuring a balanced representation of the fused data. This method builds upon the principles of uncertainty weighting [28] and extends them to the multimodal fusion context. The key novelty of the DRUW fusion method lies in its ability to dynamically adjust the weighting of each modality based on the inherent uncertainty and complementary nature of the physiological signals and speech data [29].

By collecting and analyzing data on emotional states, speech characteristics, and physiological patterns, our study aims to contribute to the development of more effective, personalized, and accessible mental health interventions. The data collected in our study can contribute to better mental health outcomes in several ways.

### Early Detection and Intervention

By correlating objective measures with subjective emotional states, we can develop tools for early detection of mental health issues, enabling timely interventions [30].

### Personalized Treatment and Monitoring

Insights from our study can inform personalized treatment plans and monitoring strategies, tailoring interventions to individual needs [31].

### Remote Monitoring and Telemedicine

Our use of wearable devices and speech analysis can contribute to remote monitoring tools, crucial for mental health support, especially in light of recent global events [32].

### Reducing Stigma and Increasing Access

By demonstrating objective measures for mental health assessment, we can potentially reduce stigma and increase access to care, particularly for underserved populations [33].

### Related Work

This section delves into various aspects of mental health monitoring research domain, including mental health data, EMA, personalization, and their real-world implications.

Recent research efforts, particularly in mental health detection and monitoring, have gained significant momentum. Key studies like the systematic review by Hickey et al [34] and Long et al [35] have critically evaluated the use of smart devices and wearable technologies. These investigations underline the capability of these devices in detecting stress, anxiety, and depression through physiological measures such as HRV, electrodermal activity, and EEG data. However, they also identify a notable gap in the availability of commercial depression-detecting devices, emphasizing the need for integrating multimodal data to enhance both accuracy and predictive power.

Recent advancements in multimodal data analysis have shown promising results in mental health diagnosis [36]. For instance, a study by Xu et al [36] proposed a measurement method for mental health based on dynamic multimodal feature recognition. This approach integrates various data sources, including physiological signals, speech patterns, and behavioral indicators, to provide a more comprehensive assessment of an individual’s mental state. Similarly, Huckins et al [37] developed a multimodal machine learning approach that combines smartphone sensing data with self-reported mental health scores to predict changes in depression and anxiety among college students.

Building on this, the role of mental health datasets becomes crucial in understanding the complex and varied nature of mental health conditions across different populations. The comprehensive analysis of datasets, such as those examined during the COVID-19 pandemic [38], offers deep insights into the mental health effects of global crises on specific demographics, like the Bangladeshi population. These datasets are instrumental not only in assessing the prevalence and severity of mental health conditions across various groups but also in supporting longitudinal studies vital for tracking changes over time.

Recent studies have also focused on improving data collection methods for mental health monitoring. For example, Morshed et al [39] introduced a novel approach using passive sensing and machine learning to predict mood instability in bipolar disorder. This method leverages smartphone usage patterns and environmental data to provide continuous, unobtrusive monitoring of mental health states.

In this study, we apply EMA [40], which represents a method for recording participants’ behavior, psychological state, and physical symptoms in real-time and at multiple time points. The primary advantage of EMA lies in its ability to minimize the biases often associated with retrospective recall in self-report data. Traditional self-report measures, which ask participants to remember and report past feelings, behaviors, or symptoms, can be influenced by memory distortions and subjective interpretations of past events, Thus, it reduces the likelihood of recall errors and increases the accuracy and reliability of the data collected.

Further, personalization in mental health monitoring systems is increasingly important [4]. Innovations in digital phenotyping [41] exemplify this trend. This is further advanced by groundbreaking approaches like those proposed by Gerczuk et al [42], using zero-shot personalization strategies for large speech foundation models in mood recognition.

The application of artificial intelligence and deep learning techniques in mental health monitoring has seen significant growth. A comprehensive review by Su et al [43] highlights the potential of deep learning models in analyzing multimodal data for mental health assessment. These advanced techniques allow for more nuanced interpretation of complex, high-dimensional data, potentially leading to more accurate and personalized mental health interventions.

In summary, the related work shows the dynamic nature of mental health detection and monitoring. However, bridging the gap between technological capabilities and personalized mental health care presents numerous challenges. The integration of multimodal data, advancements in data collection methods, and the application of sophisticated artificial intelligence techniques represent promising avenues for overcoming these challenges and improving mental health monitoring and diagnosis.

## Methods

### Methodology and Data Collection

#### Overview

The study followed a 2-week data collection protocol, during which participants wore wrist-worn devices and used a smartphone app to record their speech and self-report their emotional states. The collected data included physiological signals from the wrist-worn devices and speech recordings from the smartphone app. To collect data, we developed a platform called Mental Healthcare Internet of Things (MHIT) system.

#### The MHIT System

**Figure 1:**
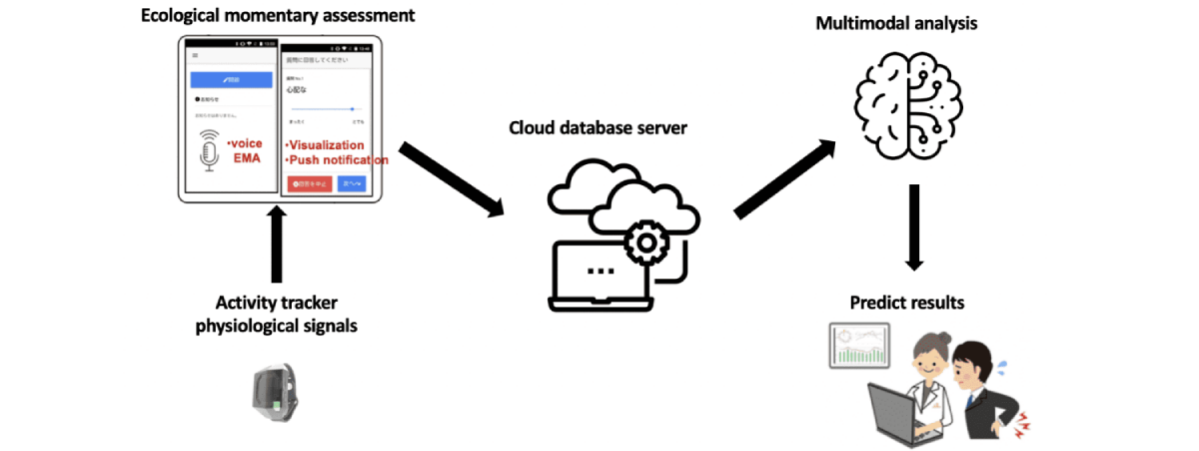
The MHIT system designed for data collection. MHIT: Mental Healthcare Internet of Things.

The MHIT system, as shown in Figure 1, is a cloud-based platform specifically crafted to gather and analyze data from Internet of Things devices. This state-of-the-art system combines the collection of physical activity signals with speech data. The MHIT system is comprised of 2 key components: a cloud server (MHIT server) and a smartphone app (MHIT app).

### Participants

A convenience sample of 298 Japanese office workers participated in our study. They were recruited by sending digital flyers. Those who agreed to participate in the study were asked to open a URL link on the flyer and complete a web-based registration form. Subsequently, the in-house wrist-worn device (ScienceNet device; ScienceNet Inc), survey progression guide, and informed consent form were mailed to them. Participants were instructed on the aim and procedure of the study through the survey progression guide. In addition, by scanning a QR code on the guide, they were able to watch a tutorial video to learn the use of the device and the MHIT app. After completing the informed consent form, they commenced the study. This recruitment process was conducted fully on the web, which contribute to achieve data collection from workers in different residential locations.

#### Annotation and EMA

This study used an EMA paradigm to capture participants’ emotional states in real time, thus avoiding potential distortions of retrospective recall in self-report data. The EMA protocol involved the following.

##### Sampling Scheme

Participants were prompted to report their emotional states by using the MHIT app at randomly selected times within –10 minutes to +10 minutes of predetermined times (11 AM, 3 PM, and 7 PM). In addition, they were instructed to voluntarily complete the same EMA questionnaires when they woke up and went to bed.

##### Data Collection

At each EMA, participants self-reported the intensity of 9 different expressed emotions on a (0:100) visual-analogue scale (slider) displayed on the MHIT app. These 9 emotions correspond to the items of the Depression and Anxiety Mood Scale (DAMS) [44]. To prevent response bias and predetermination, the order of the DAMS items was randomized for each evaluation.

#### Speech Data

Before the mental state evaluation, participants recorded their voices by speaking, for example, “the current date and time are September 5, 2022, at 10:23 PM” on the MHIT app. The reasoning behind this is to keep the content emotionally neutral. Participants also recorded activities, and the actual time was recorded by the system.

#### Physiological Signals

The instrument is fitted with a sensitive piezoelectric accelerometer that detects minute wrist accelerations (as fine as 0.01 G/rad/s), capturing even the most subtle daily movements. The ZCM within the device tallies the instances the accelerometer’s signal traverses the 0 mark over a predefined duration, known as the epoch time. Conversely, the PIM assesses the integral of the root-mean-square for the triaxial accelerometer signals. For the purposes of this investigation, we have configured the epoch interval at 1 minute, aggregating 60 data points (representing 1 hour) prior to each participant’s DAMS entry within their routine activities. To ensure the integrity of our dataset, we have meticulously curated instances that comprise both ZCM and PIM recordings, each consisting of 60 data points.

#### Annotation Scheme

The DAMS serves as a self-reported measure of an individual’s emotional state, providing a subjective assessment of their mental well-being. This scale, which encompasses nine distinct emotions—vigorous, gloomy, concerned, happy, unpleasant, anxious, cheerful, depressed, and worried—is used for comprehensively assessing mental health experiences pertinent to depression and anxiety. DAMS’s effectiveness in measuring depressive and anxious moods is particularly notable, as it uses a variety of descriptors, including adjectives, adjectival verbs, and phrases, to delineate depressive, anxious, and positive moods with high discriminant validity [44]. Moreover, its psychometric soundness has been established through methods such as parallel testing and test-retest evaluations [44], confirming its high convergent, discriminant validity, and reliability. The scale’s sensitivity to mood fluctuations is evidenced by the variance in scores observed between normal and stressful periods, underscoring its use in detecting mood changes. These features of DAMS, combined with its thorough statistical analysis across 9 emotional labels, confirmed it is a comprehensive choice for our study.

Previous research has shown that speech characteristics and patterns can reflect an individual’s emotional state. For example, depression has been associated with changes in prosody, such as reduced pitch variability and slower speaking rate [22]. Similarly, anxiety has been linked to increased vocal tension and higher fundamental frequency [45]. By analyzing speech features such as pitch, intonation, and speaking rate, we can potentially identify objective markers that correlate with the subjective emotional states reported through DAMS.

Physiological data, collected through wrist-worn accelerometers, can also provide an indirect measure of an individual’s emotional state. Studies have demonstrated that mood disorders, such as depression and anxiety, can influence an individual’s activity levels and patterns [46]. Depression, for instance, has been associated with reduced physical activity and increased sedentary behavior [47]. By examining the activity data captured by the accelerometers, we can explore potential correlations between the objective measures of physical activity and the subjective emotional states assessed by DAMS.

While speech and physical activity data do not directly measure the emotional states captured by DAMS, they can offer complementary and objective insights into an individual’s mental well-being. By combining these different modalities—self-reported mood, objective speech characteristics, and objective physiological signal patterns—we aim to develop a more comprehensive understanding of an individual’s mental health status.

#### Self-Annotation

Self-annotation is a cornerstone in daily mental health monitoring for important reasons such as capturing subjective emotional experience and ecological validity. Using the MHIT app, participants self-reported their emotional states 5 times daily over 2 weeks, using the 9 emotional states outlined in DAMS.

##### Capturing Subjective Emotional Experiences

Emotions are inherently subjective, and self-annotation allows individuals to express their emotional states based on personal experiences. This method ensures an authentic portrayal of their mental state, which is crucial for accurate mental health assessment.

##### Ecological Validity

By self-reporting in real-time within their usual environments, participants provide data that more accurately reflect their day-to-day emotional experiences, enhancing the ecological validity of our study.

### Data Preprocessing

In our research, the preprocessing of collected data was a critical step for both speech and physical activity. This process involved several stages, each tailored to the specific nature of the data being processed.

#### Preprocessing of Speech Data

For speech data, audio files were standardized in terms of their sampling rate and format for subsequent analysis.

##### Data Cleansing

Any recordings that were unsuccessful or contained data anomalies were removed. This step was crucial to ensure the integrity and quality of the speech dataset.

##### Voice Activity Detection

We used algorithms to detect and eliminate silences in voice recordings. This focus on active speech segments helped in isolating meaningful data.

##### Denoising

Background noise within the recordings was reduced using digital signal processing techniques. While the specific method may vary depending on the characteristics of the noise and the recording environment, common approaches include spectral subtraction, Wiener filtering, or more advanced techniques such as deep learning-based noise suppression algorithms [48].

#### Preprocessing of Physical Activity Data

The preprocessing of physical activity data includes the following approaches.

##### Signal Cleaning

Similar to speech data, physical activity data were cleaned to remove any erroneous signals.

##### Signal Standardization

The raw data from the physical activity sensors were standardized to ensure consistency across different participants.

#### Normalization Process

The intensity ratings of the emotional states reported by participants were normalized to a uniform scale ranging from 0 to 1.

### Multimodal Multitask Analysis

In transitioning from data collection to the analysis of daily mental health in our study, we shift our focus toward developing a robust multimodal multitask analysis framework. The initial step involves defining the analytical task, which in our case is predicting various mental health indicators as outlined by DAMS. To effectively achieve our goals in daily mental health monitoring, we need to address three pivotal questions.

- How to fuse different modalities: specifically, how do we integrate physical activities and speech data?
- How to achieve personalization: what strategies can we use to tailor the analysis to individual participants?
- How to balance different emotional states: how can we ensure that our analysis provides a balanced view of various emotional states?

To respond to these questions, our approach involves the introduction of a comprehensive framework architecture. We plan to detail each component of this framework, starting with multimodal fusion, then moving on to personalization, and concluding with multitask balancing. This sequence is carefully chosen by allowing multimodal fusion to initially integrate and align different data types (physiological signals and speech) after feature extraction, creating a whole picture of data for further analysis. Personalization subsequently adapts this integrated data to individual differences, ensuring the model accurately represents each participant’s unique mental health profile. The final stage, multitask balancing, refines the network to efficiently manage multiple analytical tasks.

### Framework

The proposed framework commences with a robust feature extraction phase. For physiological signals, PIM and ZCM are input into individual 2-layer feed-forward neural networks (FFNNs). Concurrently, speech signals are preprocessed through a specialized *wav2vec-l-emo* model [49], which is a pretrained model. These speech features are then similarly processed by a 2-layer FFNN. This standardization of feature dimensions across modalities primes the data for integration.

The fusion of these data streams is executed through a DRUW fusion block, effectively merging the standardized features from physiological signals and speech. This fusion process not only integrates the data but also applies DRUW fusion.

Upon fusion, the data advances into a specific emotional FFNN and a transformer layer, means, the combined features into a global emotional space. This space is not user-specific; rather, it serves as a shared domain, namely, macro space.

Personalization is introduced at the micro stage. Here, the framework uses additional FFNN layers tailored to individual users, enabling the selection of embedding elements pertinent to their unique emotional profiles. This adaptation leverages DRUW loss.

### DRUW Multimodal Fusion

A fundamental question in our study of mental health monitoring is “how to fuse different modalities,” specifically the integration of physical activity and speech data. To answer this, we have developed the DRUW fusion method.

The DRUW fusion formula can be represented as follows.



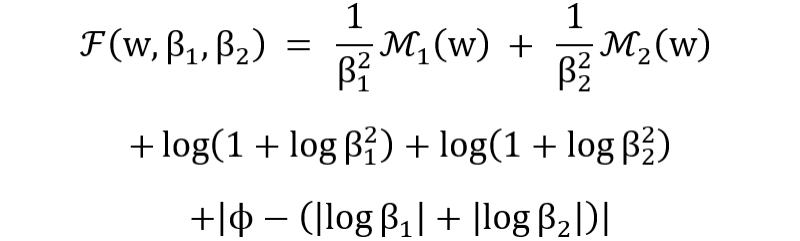



where 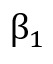 and 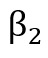 are the uncertainty parameters for the physical activity data 
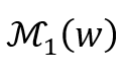
 and the speech data 
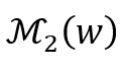
, respectively. The term 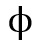 acts as a constraint, similar to 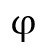 in the DRUW loss, the weighting of each modality in the fusion process is regulated, ensuring that neither modality is disproportionately represented in the fused data and maintaining a balanced integration. This adjustment, based on the uncertainty and distinct characteristics, ensures that each modality contributes appropriately to the combined dataset. Meanwhile, the complementary nature of these data types is capitalized upon by the DRUW fusion method. Physical activity data provides objective, quantifiable measures of movement and physiological responses, while speech data offers subjective insights into emotional states and mental well-being. Furthermore, a key advantage of the DRUW fusion method is its straightforward implementation.

### Macro-Micro Personalization

A key aspect of our multimodal analysis framework is the implementation of macro-micro personalization.

To quantitatively define the macro-micro personalization approach, we can formulate the integration of the macro emotional space with the micro personalization layer. This can be represented as follows.



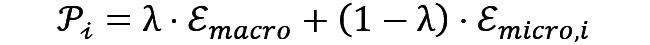



where 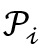 represents the personalized output for the 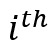 participant, 
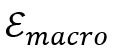
 denotes the embeddings or features extracted from the macro emotional space, 
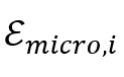
 represents the embeddings or features specific to the 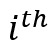 participant, and is a weighting factor that determines the balance between the influence of the macro and micro layers. It can be a fixed value or adaptively determined based on factors such as the diversity of the dataset or the specificity of the micro data.

#### Macro Emotional Space

Initially, we establish a macro emotional space that serves as a common ground for all participants. This space is built using FFNN-transformer embeddings, capturing generalized emotional patterns and trends observed across the entire participant pool. It reflects the shared aspects of emotional experiences and is crucial for understanding the broader context of mental health states.

#### Micro Personalization Space

After macro space, a micro layer is designed for each participant. This layer allows for the customization of the model based on microspecific data. It adapts the general insights from the macro space to the nuances of each participant’s emotional profile.

### DRUW Loss for Multitask

MTL is a crucial component in our study, particularly relevant to the diverse nature of mental health monitoring. MTL is a form of learning that involves training a model on several related tasks simultaneously. This approach is underpinned by the principle that “transfer should always be useful”; essentially, any pair of tasks should share some commonalities in their underlying distributions [50].

In addressing the critical challenge of “how to balance different emotional states” in our study, we use the DRUW loss [29], a solution developed in our previous work. This approach is particularly crucial in the context of multitask learning, where balancing the contribution of each task—especially when dealing with a spectrum of emotional states—is key to the overall model’s performance. This method allows for the adaptive balancing of tasks, taking into account the varying degrees of complexity and uncertainty inherent in each task. The equation of the DRUW loss function is as follows.



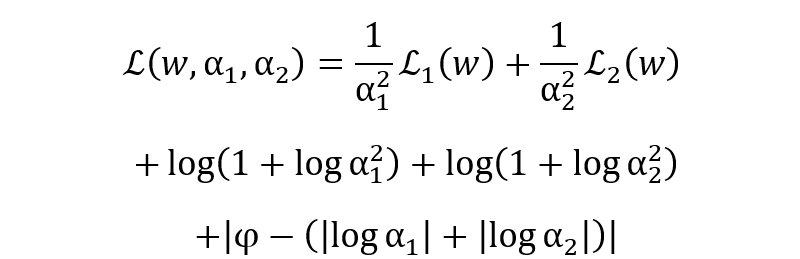



Where 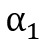 and 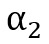 are uncertainty parameters corresponding to different tasks in our model, with 
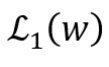
 and 
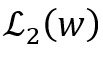
 representing the respective task-specific loss functions. The inclusion of 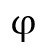 serves as a constraint, regulating the sum of these weights to prevent trivial solutions and maintain the balance among tasks.

### Evaluation

#### Experimental Setup

To construct a reliable evaluation scheme, the dataset is partitioned into training, development, and test sets based on time-dependent criteria, as outlined in Table 1. Given that the dataset is collected over a 2-week period, we allocate the first 70% of the data from each participant to the training set. The subsequent 15% forms the development set, and the remaining 15% constitutes the test set. This partitioning strategy ensures that the evaluation is robust and reflects the temporal dynamics of the data.

**Table 1:** Data partitioning.

Set	Data (%)	Samples
Training	70	4340
Development	15	931
Test	15	929

#### Evaluation Metrics

In this study, the concordance correlation coefficient (CCC) is used as a key evaluation metric. CCC considers both the scale and location shifts between the predicted and actual data, providing a comprehensive measure of the model’s predictive performance. In our context, this metric is crucial for assessing the accuracy of our model in reflecting the true emotional experiences of participants.

The CCC is defined as follows.



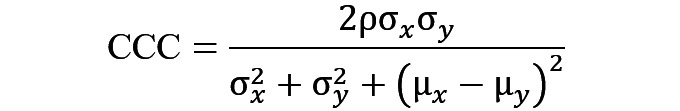



Where 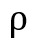 represents the Pearson correlation coefficient between the predicted and actual values, 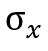 and 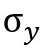 are the SDs of the predicted and actual values, respectively, and 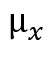 and 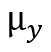 are their means.

#### Benchmark Model

To effectively evaluate our macro-micro framework, it is crucial to establish a benchmark for comparison. This benchmark model consists of the following key components: pure concatenation for modality fusion, 2-layer FFNN for presentation, and equal weight strategy for MTL. This benchmark model, with its straightforward concatenation, basic personalization, and uniform task weighting, provides a solid foundation for comparison.

##### Pure Concatenation for Modality Fusion

This approach linearly combines features from both physical activity and speech data without weighting or transformation.

##### 2-Layer FFNN for Personalization

We designed FFNN to adapt the concatenated features to get personalized embeddings.

##### Equal Weight Strategy for MTL

In handling multiple tasks, we applied an equal weight strategy across all tasks.

### Comparison Methods

#### Multimodal Fusion Techniques

In our exploration of multimodal fusion techniques, we first investigated the use of separate transformer embeddings for each modality. This approach aimed to capture unique features within physical activity and speech data independently before combining them. The results indicate that while this method was effective in isolating modality-specific characteristics, it also necessitated sophisticated alignment strategies during the fusion stage.

We also applied attention mechanisms, including solo attention [51] and postconcatenation attention. These techniques allowed the model to dynamically focus on the most informative features from each modality. The solo attention mechanism, applied before concatenation, proved particularly effective in enhancing the model’s sensitivity to contextually relevant multimodal cues.

A more straightforward approach, the pure weighted method, involved assigning fixed weights to each modality during fusion. Despite its simplicity, this method displayed limitations in adaptability, especially in scenarios where the relative importance of each modality varied.

Besides, max fusion [52] was used to capture the most significant features across modalities by taking the maximum value across feature dimensions. This method was found to be particularly useful in scenarios where the dominant features in the data were more predictive of the outcome.

We tested gated fusion [53], which was designed for dynamic control over the contribution of each modality based on the data’s contextual information. This adaptability resulted in improved performance, especially in complex scenarios where the relevance of each modality changed.

#### Personalization Strategies

The FFNN was used as a baseline for personalization, adapting concatenated multimodal features to individual profiles. Comparatively, we used a transformer model without separate emotional FFNN for personalization.

We also applied an adapter [54] to compare. The adapter method involved integrating small, trainable modules into a pretrained model. This approach facilitated efficient and effective personalization without the need for extensive retraining.

#### MTL Approaches

**Figure 2:**
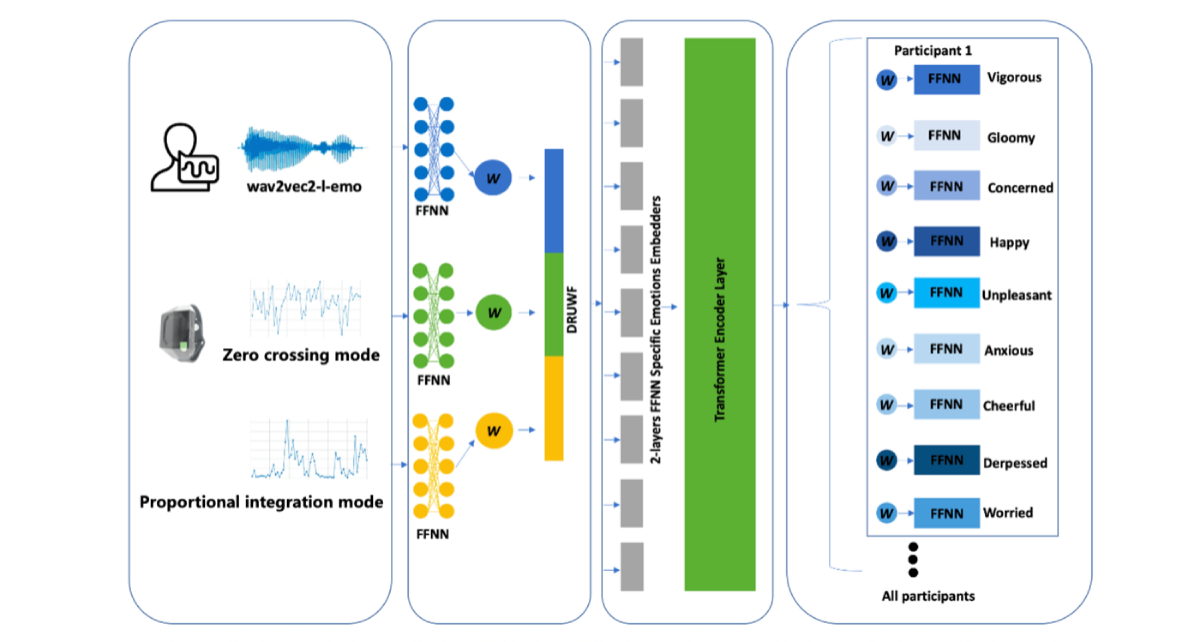
Macro-micro personalization framework for multimodal-multitask learning in mental health monitoring. W: weight; DRUW: Dynamic Restrained Uncertainty Weighting; DRUWF: Dynamic Restrained Uncertainty Weighting Fusion; FFNN: feed-forward neural network. The framework integrates physiological signals and speech data through feature extraction, DRUWF, macro layers with transformer encoder, and micro personalized layers.

Using a single embedding to produce outputs for multiple tasks with equal weighting provided a baseline for multitask performance. We also compared the pure weighted approach, assigning fixed weights to each task, which showed some improvements over the equal weight strategy but still lacked the dynamic adaptability required for more complex multitask scenarios. The architecture of the proposed model is given in Figure 2.

### Model Training Procedure

All models in our study were trained over 100 epochs using stochastic gradient descent with an initial learning rate set at 0.001, using a Nesterov momentum of 0.9 to enhance convergence. The learning rate was adaptively reduced by a factor of 0.9 if no improvement was observed on the development set after 5 consecutive epochs, ensuring efficient optimization. The training was conducted with a batch size of 16, balancing computational resources and model performance. Additionally, a weight decay of 0.0001 was applied to prevent overfitting. The final model configuration selected for evaluation on the test set was the one yielding the best performance on the development set, ensuring the reliability and robustness of our results. Before producing the final output, we used a sigmoid function to ensure that the predicted values ranged from 0 to 1. This adjustment was necessary because our labels had been normalized to a scale of 0 to 1.

### Ethical Considerations

This study was approved by the Ethics Committee of the University of Tokyo (21-353). The study participants provided written informed consent.

## Results

### Overview

Our results first show CCC for various emotional dimensions using different single-modal data types (physical activity and audio), with and without personalization in Table 2. Our analysis also investigated the efficacy of various multimodal fusion techniques and their capacity for personalization in assessing different emotional dimensions in Table 3. The multimodal fusion approaches examined included basic, max fusion, gated fusion, attention fusion, solo-attention fusion, cross-modal attention, and a number of proposed methods. Each of these techniques was also analyzed in conjunction with various multitasking frameworks such as basic, transformer, adapter, equal, multioutputs, and our proposed method. In comparison between the 2 tables, multimodal results generally outperform single-modal ones.

The results indicate a differential impact on the CCC across emotional states and fusion methods. For instance, the max fusion approach yielded a CCC of 0.451 for vigorous, which was a notable improvement over the basic approach’s 0.415. However, this method seemed less effective for gloomy, with a CCC of 0.356. In contrast, the gated fusion technique exhibited a more consistent performance across different emotional states, with CCCs ranging from 0.277 for anxious to 0.537 for worried.

Of particular interest were the results from the proposed methods, which showed promising CCC values across several emotional states. The highest recorded CCC of the proposed methods was for worried, with a value of 0.581. Conversely, gloomy showed the lowest CCC at 0.377 using the same proposed methods.

Overall, the mean CCC values across all emotional states suggested that the proposed methods combined with our proposed multitasking framework outperformed the other techniques, achieving a mean CCC of 0.503. This mean value was computed by averaging CCCs across all the emotional states for each method. Notably, the proposed methods consistently yielded CCC values above the overall mean, underscoring their potential for enhancing emotion recognition tasks in multimodal settings.

In conclusion, our results underscore the importance of choosing the appropriate fusion and multitasking methods to maximize the agreement between predicted and actual emotional states. The proposed methods, when tailored for individual emotional dimensions, demonstrate significant promise for personalization, which is a critical aspect of effective emotional state prediction.

**Table 2:** CCC^a^ for various emotional dimensions using different single-modal data types (physical activity and audio), with and without personalization. The emotional dimensions covered are vigorous, gloomy, concerned, happy, unpleasant, anxious, cheerful, depressed and worried.

Single model and personalization			Vigorous		Gloomy		Concerned		Happy		Unpleasant		Anxious		Cheerful		Depressed		Worried		Mean (SD)^b^
**ZCM^c^**																					
	No	0.287		0.187		0.325		0.341		0.364		0.179		0.224		0.282		0.334		0.281 (0.069)	
	Yes	0.407		0.426		0.499		0.485		0.487		0.349		0.322		0.454		0.535		0.441 (0.071)	
**PIM^d^**																					
	No	0.130		0.142		0.368		0.251		0.374		0.244		0.006		0.302		0.291		0.225 (0.121)	
	Yes	0.145		0.315		0.499		0.368		0.489		0.272		0.051		0.493		0.537		0.341 (0.172)	
**Speech**																					
	No	0.287		0.187		0.325		0.341		0.364		0.179		0.224		0.282		0.334		0.281 (0.069)	
	Yes	0.407		0.426		0.499		0.485		0.487		0.349		0.322		0.454		0.535		0.441 (0.071)	

^a^CCC: concordance correlation coefficient.

^b^The “Mean” column represents the average CCC across the emotional dimensions.

^c^ZCM: zero crossing mode.

^d^PIM: proportional integration mode.

**Table 3:** CCC^a^ for various emotional dimensions, measured using participant-dependent partitions on our dataset. The table also demonstrates the effectiveness of different fusion methods and indicates whether personalization was used. The emotional dimensions covered are vigorous, gloomy, concerned, happy, unpleasant, anxious, cheerful, depressed, and worried.

Multimodal fusion	Personalization	Multitask	Vigorous	Gloomy	Concerned	Happy	Unpleasant	Anxious	Cheerful	Depressed	Worried	Mean (SD)^b^
Basic	Basic	Basic	0.415	0.404	0.469	0.452	0.499	0.212	0.304	0.472	0.514	0.416 (0.099)
Max fusion	Basic	Basic	0.451	0.356	0.464	0.465	0.501	0.356	0.325	0.421	0.509	0.428 (0.067)
Gated fusion	Basic	Basic	0.417	0.417	0.495	0.473	0.497	0.277	0.317	0.446	0.537	0.431 (0.086)
Attention fusion	Basic	Basic	0.414	0.281	0.524	0.413	0.554	0.386	0.317	0.447	0.574	0.435 (0.102)
Solo-attention fusion	Basic	Basic	0.403	0.337	0.488	0.467	0.513	0.394	0.298	0.470	0.540	0.434 (0.082)
Cross-modal attention	Basic	Basic	0.436	0.443	0.483	0.455	0.497	0.268	0.323	0.463	0.527	0.447 (0.084)
Proposed	Basic	Basic	0.393	0.401	0.504	0.435	0.488	0.469	0.322	0.465	0.556	0.449 (0.069)
Basic	Transformer	Basic	0.434	0.330	0.547	0.470	0.548	0.538	0.325	0.421	0.578	0.466 (0.09)
Basic	Adapter	Basic	0.457	0.329	0.554	0.463	0.549	0.543	0.320	0.432	0.586	0.472 (0.098)
Basic	Proposed	Basic	0.460	0.401	0.554	0.462	0.544	0.557	0.351	0.441	0.585	0.484 (0.080)
Basic	Basic	Equal	0.420	0.321	0.521	0.436	0.511	0.446	0.298	0.381	0.563	0.431 (0.090)
Basic	Basic	Multioutput	0.407	0.399	0.493	0.470	0.526	0.469	0.324	0.467	0.531	0.454 (0.067)
Basic	Basic	Proposed	0.434	0.330	0.547	0.470	0.548	0.538	0.325	0.422	0.579	0.466 (0.095)
Proposed	Proposed	Basic	0.414	0.389	0.564	0.522	0.556	0.581	0.364	0.419	0.589	0.489 (0.091)
Basic	Proposed	Proposed	0.454	0.450	0.549	0.519	0.554	0.581	0.358	0.424	0.583	0.497 (0.079)
Proposed	Basic	Proposed	0.449	0.377	0.525	0.466	0.554	0.573	0.296	0.437	0.543	0.469 (0.091)
Proposed	Proposed	Proposed	0.464	0.464	0.549	0.538	0.554	0.581	0.373	0.420	0.581	0.503 (0.075)

^a^CCC: concordance correlation coefficient.

^b^The “Mean” column represents the average CCC across the emotional dimensions.

### Statistical Validation

We also conducted a statistical analysis to complement the CCC results from our deep learning model, crucial for validating the model’s reliability and generalizability across different datasets and conditions. Our mixed linear model analysis [55], presented in Table 4, reveals 2 critical insights. First, the highly significant within-individual associations (Q) across 9 emotional scales underscore the model’s capability to capture nuanced emotional responses, indicating its robust predictive power. Second, the observation of group and residual variances highlights the variability that the model does not account for, signaling areas that require further refinement. This unexplained variability invites a deeper investigation into potential factors, such as the model’s sensitivity to specific data characteristics or the necessity for incorporating a more diverse range of training data. Understanding these elements can guide targeted improvements in the model’s architecture and training process, ultimately enhancing its accuracy and applicability in personalized mental health monitoring.

**Table 4:** Mixed linear model regression results for emotional dimensions^a^.

Emotional dimension	Intercept	Regression coefficient	SE	*z* score	*P* value>|*z*|	95% CI	Group variance	Residual variance
Vigorous	0.155	0.549	0.076	7.176	<.001	0.399-0.698	0.039	0.057
Gloomy	0.087	0.607	0.064	9.512	<.001	0.482-0.733	0.023	0.067
Concerned	0.153	0.500	0.073	6.813	<.001	0.356-0.644	0.043	0.057
Happy	0.236	0.446	0.077	5.771	<.001	0.295-0.598	0.030	0.064
Unpleasant	0.071	0.630	0.066	9.534	<.001	0.501-0.760	0.028	0.061
Anxious	0.127	0.577	0.071	8.177	<.001	0.439-0.716	0.035	0.061
Cheerful	0.210	0.477	0.074	6.475	<.001	0.333-0.621	0.022	0.072
Depressed	0.044	0.647	0.060	10.758	<.001	0.529-0.765	0.021	0.062
Worried	0.138	0.539	0.076	7.097	<.001	0.390-0.688	0.048	0.052

^a^The table summarizes the intercept, regression coefficients, standard errors, *z* scores, *P* values, CIs, group variances, and residual variances for each emotion studied. The emotional dimensions covered are vigorous, gloomy, concerned, happy, unpleasant, anxious, cheerful, depressed, and worried.

## Discussion

### Overview

This study introduces a novel dataset and a macro-micro framework for personalized daily mental health monitoring, leveraging multimodal and MTL strategies. The results demonstrate the efficacy of our approach in predicting emotional states, with a mean CCC of 0.503 across 9 emotional dimensions.

The proposed macro-micro framework, which combines macro-level emotion transformer embeddings with micro-level personalization layers, shows superior performance compared to traditional approaches. This suggests that incorporating both general emotional patterns and individual-specific adaptations is crucial for accurate mental health monitoring. The effectiveness of our DRUW fusion method in integrating multimodal data further underscores the importance of adaptive weighting strategies in handling diverse data types.

Our findings align with previous studies highlighting the potential of multimodal approaches in mental health monitoring. However, our work extends beyond existing research by incorporating personalization at both macro and micro levels, addressing a critical gap in current mental health technology.

The high significance of within-individual associations across emotional scales, as revealed by our mixed linear model analysis, validates the model’s capability to capture nuanced emotional responses. This has important implications for the development of personalized mental health interventions, as it suggests that our model can detect subtle changes in an individual’s emotional state over time.

However, the observed group and residual variances in our statistical analysis indicate that there is still unexplained variability in emotional states. This highlights a limitation of our model and suggests that additional factors, not captured in our framework, may influence daily emotional states. These could include external stressors, social interactions, or physiological factors not measured in our study.

Another limitation is the reliance on self-reported emotional states, which, while valuable for capturing subjective experiences, may be subject to reporting biases. Future research could explore the integration of objective measures of emotional state, such as facial expression analysis or additional physiological markers, to complement self-reports.

Looking ahead, several avenues for future research emerge from our findings. First, expanding the dataset to include a more diverse range of participants and longer monitoring periods could enhance the generalizability of our model. Second, investigating the incorporation of additional modalities, such as sleep patterns or social media activity, could provide a more comprehensive picture of mental health. Finally, exploring the application of our framework in clinical settings could help bridge the gap between research and practical mental health interventions.

### Conclusions

In conclusion, this study introduces a groundbreaking dataset and a macro-micro framework that significantly advances personalized daily mental health monitoring. By leveraging multimodal and MTL strategies, we have demonstrated a robust model capable of predicting emotional states. The statistical analysis further validates the model’s reliability, highlighting its potential for wider application in the mental health domain. Moving forward, our focus will be on expanding the dataset, incorporating additional modalities, and refining our model to address these variances, with the ultimate goal of making daily mental health monitoring a more accessible, nonintrusive, and personalized practice.

## Abbreviations

CCC: concordance correlation coefficient
DAMS: Depression and Anxiety Mood Scale
DRUW: Dynamic Restrained Uncertainty Weighting
EEG: electroencephalography
EMA: ecological momentary assessment
FFNN: feed-forward neural network
HRV: heart rate variability
MHIT: Mental Healthcare Internet of Things
MTL: multitask learning
PIM: proportional integration mode
ZCM: zero crossing mode.
